# Alcohol in Medicine

**Published:** 1889-11-16

**Authors:** 


### ALCOHOL IN MEDICINE.

In the New York *Medical Journal* for September 28th last, Dr. J. M. Farrington discourses on the use of alcohol in medicine. The tide of evils that flow from alcohol has been recognised and acknowledged by every unprejudiced observer. As conservator of the lives of fellow-creatures, it behoves the physician to most seriously consider before "unchaining the tiger" alcohol. Dr. Farrington earnestly protests against the teaching of most recognised authorities on the subject, and he quotes Dr. Davis, of Chicago, who has said, "step by step the progress of science has nullified every theory on which the physician administers alcohol." But Dr. Farrington does not give us any new reasons for abolishing alcohol as a medicine. He quotes a number of authorities both for and against, as Bartholow, Richardson, Ringer, Bidwell, the United States Dispensatory, etc., but on the whole we are inclined to think the authorities he quotes are decidedly in favour of the judicious use of alcohol in medicine. It is curious that those who would not hesitate to use ammonia or ether as a stimulant in medicine should still decline alcohol. Yet alcohol in some shape or form is generally the most easily obtained stimulant, and we certainly have seen many persons who would have died had alcohol not been administered.

